# Risk Factors of *Coxiella burnetii* (Q Fever) Seropositivity in Veterinary Medicine Students

**DOI:** 10.1371/journal.pone.0032108

**Published:** 2012-02-21

**Authors:** Myrna M. T. de Rooij, Barbara Schimmer, Bart Versteeg, Peter Schneeberger, Boyd R. Berends, Dick Heederik, Wim van der Hoek, Inge M. Wouters

**Affiliations:** 1 Division of Environmental Epidemiology, Institute for Risk Assessment Sciences, Utrecht, the Netherlands; 2 Centre for Infectious Disease Control, National Institute for Public Health and the Environment, Bilthoven, the Netherlands; 3 Department of Medical Microbiology and Infection Control, Jeroen Bosch Hospital, ‘s-Hertogenbosch, the Netherlands; 4 Division of Veterinary Public Health, Institute for Risk Assessment Sciences, Utrecht, the Netherlands; University of São Paulo, Brazil

## Abstract

**Background:**

Q fever is an occupational risk for veterinarians, however little is known about the risk for veterinary medicine students. This study aimed to assess the seroprevalence of *Coxiella burnetii* among veterinary medicine students and to identify associated risk factors.

**Methods:**

A cross-sectional study with questionnaire and blood sample collection was performed among all veterinary medicine students studying in the Netherlands in 2006. Serum samples (n = 674), representative of all study years and study directions, were analyzed for *C. burnetii* IgG and IgM phase I and II antibodies with an immunofluorescence assay (IFA). Seropositivity was defined as IgG phase I and/or II titer of 1∶32 and above.

**Results:**

Of the veterinary medicine students 126 (18.7%) had IgG antibodies against *C. burnetii*. Seropositivity associated risk factors identified were the study direction ‘farm animals’ (Odds Ratio (OR) 3.27 [95% CI 2.14–5.02]), advanced year of study (OR year 6: 2.31 [1.22–4.39] OR year 3–5 1.83 [1.07–3.10]) having had a zoonosis during the study (OR 1.74 [1.07–2.82]) and ever lived on a ruminant farm (OR 2.73 [1.59–4.67]). Stratified analysis revealed study direction ‘farm animals’ to be a study-related risk factor apart from ever living on a farm. In addition we identified a clear dose-response relation for the number of years lived on a farm with *C. burnetii* seropositivity.

**Conclusions:**

*C. burnetii* seroprevalence is considerable among veterinary medicine students and study related risk factors were identified. This indicates Q fever as an occupational risk for veterinary medicine students.

## Introduction

Q fever is a zoonotic disease caused by the bacterium *Coxiella burnetii* and is, apart from community outbreaks, known as an occupational disease of veterinarians, farmers and abattoir workers [1]. Symptomatic acute Q fever mainly presents as fever and headache, hepatitis, or pneumonia [2], [3]. Moreover, infection with *C. burnetii* is asymptomatic in approximately 60% of those infected [2]. Many Q fever infections are not diagnosed because of the often mild and nonspecific clinical symptoms [4]. Acute Q fever, whether or not symptomatic, can develop into chronic Q fever [3]. Chronic Q fever generally presents as a culture-negative endocarditis or vascular infection with a high case fatality [3]. Another important long-term effect is Q fever fatigue syndrome, which occurs in 10 to 20% of all acute Q fever cases [5].

*C. burnetii* is a pathogenic bacterium which can infect mammals, birds and arthropods [1]. Transmission of *Coxiella* to humans occurs primarily through air via bioaerosols [6]. Furthermore humans can be infected by intake of contaminated milk or food, but these routes of transmission are of minor relevance [7]. The *Coxiella* bacterium is known to have two antigenic stages: the virulent phase I variant and the avirulent phase II variant [8]. In the body, *C. burnetii* is controlled by the T-cell dependent immune system, resulting in the production of specific antibodies [2]. Immunoglobulin G (IgG) is primarily effective against phase II antigen, while Immunoglobulin M (IgM) targets both phase I and II antigens [2]. The level of IgM increases rapidly after infection, thus is considered to be a marker of recent infection, however it can persist for many months [9], [10]. IgG levels increase a few weeks after infection, but remain detectable for years or even throughout life [5], [9].

Before the large community outbreaks in the Netherlands starting in 2007, *C. burnetii* seroprevalence was 2.4% in a general population sample taken in 2006–2007 [11]. Furthermore the study showed that persons who kept ruminants or with occupational animal contact had a higher risk to be infected with *Coxiella* [11]. Serum samples collected in the Netherlands in November 2009 showed that more than half of the livestock veterinarians were seropositive [12]. A similarly high seroprevalence for *C. burnetii* in veterinarians has been reported in other studies, with prevalence ranging from approximately 20 to 50% [13], [14]. Hence a substantial number of veterinarians become infected during their career, or possibly during their veterinary education. Veterinary medicine students perform similar activities as veterinarians during their study and likely have an increased risk to become infected with *C. burnetii* also. Yet, little is known about seroprevalence among veterinary students and the possible risk factors.

Few serological studies have been done among veterinary students, showing prevalence figures of *Coxiella* antibodies to range from 10 to 40% [15]–[17]. Valencia *et al* showed that students at the beginning of their first study year had a seroprevalence of 4.0% which was significantly lower compared to the 16.8% prevalence in the fifth year, implying a gradual increase in prevalence over the study periods [16]. However, studies reporting on the seroprevalence for *C. burnetii* covering the complete educational program and study duration are thus far missing. In univariate analysis some risk factors for seropositivity were identified in these studies, i.e. male gender, contact with ruminants, and study direction, although multivariate analyses were not carried out [16], [17]. We thus performed a large-scale cross sectional study to determine the seroprevalence of *C. burnetii* among all veterinary medicine students studying in the Netherlands in the year 2006. All study years and study directions were included in order to identify the pattern in seroprevalence of antibodies against *C. burnetii* and to determine the associated study-related factors and other student characteristics.

## Methods

### Study design and population

The cross sectional design and study population have been described before by Samadi *et al* [18]. Briefly, all 1416 students, who were registered as a student of veterinary medicine in 2006 at Utrecht University, the only faculty of Veterinary Medicine in the Netherlands, were requested to participate. Students of all study phases were asked to fill in an online questionnaire and were invited to donate a blood sample of 20 ml for serological testing. Non-responders were sent maximally two reminders. Blood collection was performed in 2006 before the start of large community outbreaks of Q fever in the Netherlands in 2007–2009.

### Ethics statement

The study protocol was approved by the Ethical Committee of the Utrecht University. All participants gave written informed consent prior to blood collection.

### Questionnaire

Information was collected on participants' demographic and study related characteristics and on their smoking habits and health status. Regular contact with diverse animal species was asked for during different periods of childhood and adulthood. Information was gathered about a farm childhood, the number of years lived on a farm, farm type and the activities performed on the farm. Questions about health status addressed general health, clinical symptoms and self-reported zoonotic diseases.

Study related characteristics for veterinary medicine students in the Netherlands are affected by the structure of the veterinary curriculum with its variety of directions and theoretical/practical stages. Six months after the start of the study the veterinary curriculum divides into two main directions: ‘individually kept animals’ and ‘farm animal health’. After the second study year, the curriculum subdivides further. The direction ‘individually kept animals’ is split into ‘companion animals’ and ‘equine’. The direction: ‘farm animal health’ is also split further in ‘farm animals and veterinary public health’ and ‘veterinary scientific research’. The first two study years consist of theoretical courses. During the third and fourth year the content of the courses shifts gradually towards practical lessons, but the majority is still theoretical. Fifth-year students start to follow internships at all departments but with the emphasis on their own specialization. Students with the companion animal direction mostly encounter cats and dogs, students at the equine department focus on horses and students doing the farm animal health specialization encounter mainly cows, pigs, poultry, sheep and goats.

### Detection of *C. burnetii* IgG and IgM

Sera were analyzed for phase I and phase II IgG antibodies against *C. burnetii* at the Regional Laboratory of Medical Microbiology and Infection Control of the Jeroen Bosch Hospital in Den Bosch, using an Immunofluorescence Assay (IFA) according to the manufacturer's protocol (Focus Diagnostics). Sera were tested in a dilution series starting from a 1∶32 till a 1∶4096 dilution. An antibody titer of 1∶32 and above for either IgG I or II antibodies of a serum sample was defined seropositive. A positive IgG test was followed by determination of phase I and II IgM antibodies by IFA.

### Statistical analysis

All statistical analyses were carried out using SPSS for Windows (version 16). Univariate regression analyses were performed to investigate the association between seropositivity and possible risk factors. Variables in univariate analysis associated with seropositivity (p<0.20) were selected for multivariate logistic regression analyses. These variables were tested for multicollinearity and after assumptions were met, both forward and backward regression analyses were applied. The final multivariate model was obtained with the criteria of a p-value of less than 0.05 for the model and for each variable itself. Smoothed regression analysis was performed to assess the shape of the association between seropositivity and the number of years a student had lived on a farm.

## Results

### Response

**Figure 1:**
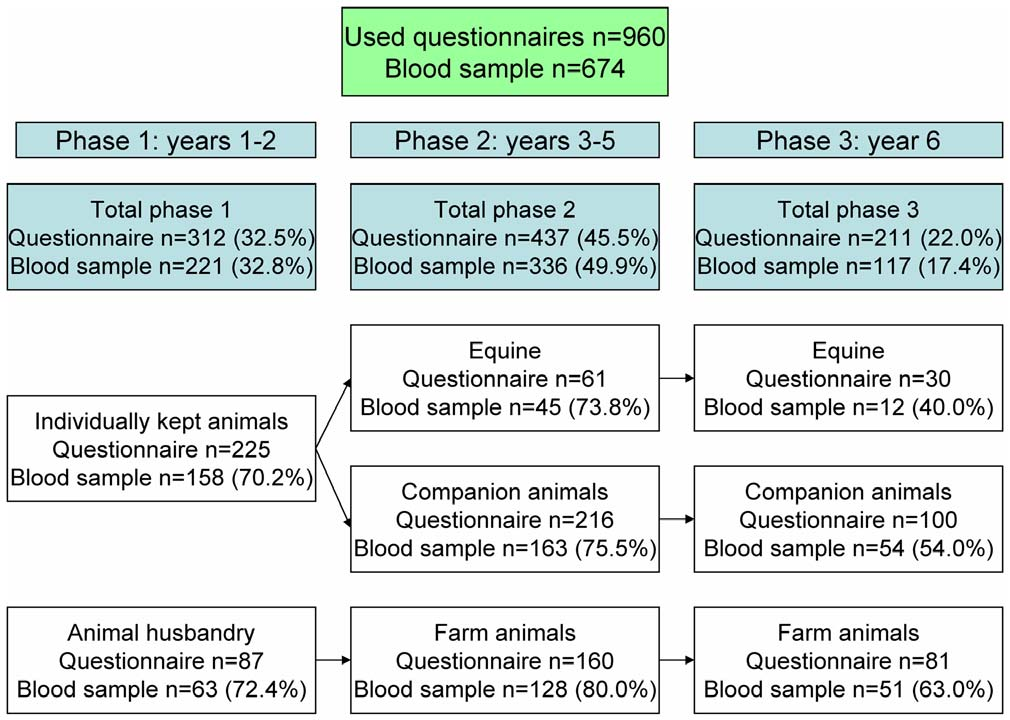
Numbers and percentages of participants per study direction and study phase.

In total, 965 of all the 1416 veterinary medicine students responded to the questionnaire (68.2%) of which 5 were excluded in further analyses. One student was excluded because the questionnaire was not completed and four others as they represented study specializations with intrinsic low numbers. Of the 960 students providing a questionnaire, 674 students provided a blood sample as well (47.6% of the total population). The division over the different study phases and study directions of the respondents is shown in Figure 1.

### Participants' characteristics

Of the participants that completed the questionnaire, 80% were women (Table 1). The mean age was 24 years with a range from 18 to 47 years. A high number (51.1%) of the students reported previous or current regular contact with farm animals outside the veterinary curriculum. Furthermore 645 students (67.2%) had regular contact with horses and 97.6% of the students had regular contact with pets. Of the students 39.5% grew up in a rural area and 13.5% had ever lived on a farm. Demographic characteristics of students who did not provide blood were generally similar to those who did, except for borderline significance for having lived on a farm or in a village (Table 1). Of the students 130 reported to have had a zoonosis during their study of which were reported most frequently: dermatophytosis (ringworm, 8.5%) and other fungal infections (5.5%, Table 2).

**Table 1:** Descriptive characteristics (n (%) or stated otherwise) of the total study population and those who did and did not provide a blood sample.

Population characteristics	total	with blood	without blood
Number of students	960	674	286
Female	771 (80.3%)	540 (80.1%)	231 (80.8%)
Age, AM^a^ (SD^b^)	23.7 (3.7)	23.7 (3.6)	23.9 (3.8)
Weight (kg), AM^a^ (SD^b^)	68.5 (11.2)	68.3 (10.7)	69.1 (12.3)
Height (cm), AM^a^ (SD^b^)	174.6 (8.3)	174.4 (8.2)	175.2 (8.5)
Current smoker	103 (10.7%)	69 (10.2%)	34 (11.8%)
Past Smoker	86 (8.9%)	60 (8.9%)	26 (9.0%)
Regular contact^c^ with animals besides the study:			
Horses	645 (67.2%)	451 (66.9%)	194 (67.8%)
Cows	312 (32.5%)	216 (32.0%)	96 (33.6%)
Pigs	136 (14.2%)	94 (13.9%)	42 (14.7%)
Sheep	275 (28.6%)	192 (28.5%)	83 (29.0%)
Poultry	307 (32.0%)	220 (32.6%)	87 (30.4%)
Goats	232 (24.2%)	166 (24.6%)	66 (23.1%)
Dogs	717 (74.7%)	507 (75.2%)	210 (73.4%)
Cats	712 (74.2%)	496 (73.6%)	216 (75.5%)
Rodents	715 (74.5%)	505 (74.9%)	210 (73.4%)
Birds	394 (41.0%)	283 (42.0%)	111 (38.8%)
Job with previous or current regular animal contact	439 (45.7%)	307 (45.5%)	132 (46.2%)
Growing up in rural area (village)^d^	379 (39.5%)	282 (41.8%)	97 (33.9%)
Farm childhood^e^	130 (13.5%)	100 (14.8%)	30 (10.5%)
Self reported zoonosis during VM^f^	190 (19.8%)	132 (19.6%)	58 (20.3%)
Self reported Q fever	0 (0%)	0 (0%)	0 (0%)
Positive Q fever status		126 (18.7%)	

a AM, Arithmetic Mean.

b SD, Standard Deviation.

c Previous or current regular contact (>once a week).

d Chi-square between providing and not-providing blood borderline significant with p = 0.07.

e Chi-square between providing and not-providing blood borderline significant with p = 0.08.

f VM, veterinary medicine.

**Table 2:** Overview of self-reported zoonotic diseases reported by veterinary medicine students (n = 960) during the veterinary medicine study.

Self reported zoonoses during VM^a^	Number (%)
Brucellosis	0 (0%)
Campylobacteriosis	10 (1.5%)
Cryptosporidiosis	0 (0%)
Ecthyma	9 (1.3%)
Giardiasis	1 (0.1%)
Cat scratch	3 (0.4%)
Leptospirosis	0 (0%)
Listeriosis	2 (0.3%)
Psittacosis	0 (0%)
Q fever	0 (0%)
Salmonellosis	8 (1.2%)
Dermatophytosis (ringworm)	57 (8.5%)
Other fungal infections	37 (5.5%)
Staphylococcus	5 (0.7%)
Toxoplasmosis	0 (0%)
VTEC	2 (0.3%)
Worminfection	13 (1.9%)

a VM, veterinary medicine.

### Serological results

Sera of 126 students (18.7%) were positive, with an IgG II titer ranging from 1∶32 to 1∶4096. Thirty percent (n = 38) of the students with a positive IgG II titer also had a positive IgG I titer ranging from 1∶32 to 1∶2084. There were no students with exclusive positive IgG I titers. Only sera with a positive IgG titer were tested for IgM antibodies. Of the IgG positives, 3% also had a positive IgM I with titers ranging from 1∶32 to 1∶256. While 19% of the IgG positives had also a positive IgM II indicating recent infection, with titers from 1∶32 to >1∶256. Seroprevalence showed an increase from study phase 1 (year 1–2) to phase 2 (year 3–5) and to phase 3 (year 6). Additionally, students mostly involved with farm animals had a much higher seroprevalence than those working with individually kept animals (Table 3).

**Table 3:** Characteristics of students (n (%) or stated otherwise) who provided blood for the different study phases and by study direction.

*Students study phase 1 (Year 1–2)*	Farm animals	Individually kept animals
Number of students	63	158
Contact with ruminants outside VM^a^	44 (69.8%)	43 (27.2%)
Job with regular animal contact	29 (46.0%)	72 (45.6%)
Growing up in rural area (village)	38 (60.3%)	52 (32.9%)
Farm childhood	17 (27.0%)	16 (10.1%)
Positive *C. burnetii* status	15 (23.8%)	9 (5.7%)

*Students study phase 2 (Year 3–5)*	Farm animals	Companion animals	Horse
Number of students	128	163	45
Contact ruminants outside VM^a^	95 (74.2%)	48 (29.4%)	18 (40%)
Job with regular animal contact	57 (44.5%)	65 (39.9%)	29 (64.4%)
Growing up in rural area (village)	61 (47.7%)	59 (36.2%)	21 (46.7%)
Farm childhood	40 (31.2%)	10 (6.1%)	5 (11.1%)
Positive *C. burnetii* status	46 (35.9%)	19 (11.7%)	6 (13.3%)

*Students study phase 3 (Year 6)*	Farm animals	Companion animals	Horse
Number of students	51	54	12
Contact with ruminants outside VM^a^	27 (52.9%)	15 (27.8%)	6 (50%)
Job with regular animal contact	22 (43.1%)	27 (50.0%)	6 (50.0%)
Growing up in rural area (village)	24 (47.1%)	19 (35.2%)	8 (66.7%)
Farm childhood	7 (13.7%)	3 (5.6%)	2 (16.7%)
Positive *C. burnetii* status	19 (37.3%)	10 (18.5%)	2 (16.7%)

Note.

a Previous or current regular (>once a week) contact with ruminants outside the veterinary medicine curriculum.

### Risk factor analyses

**Figure 2:**
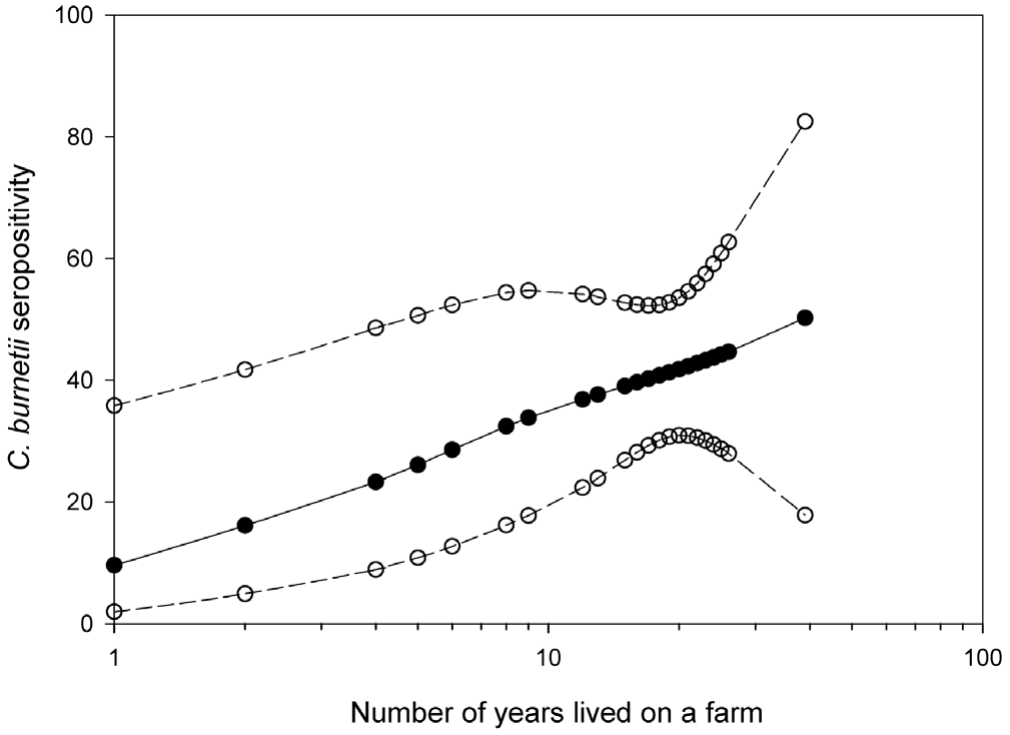
Association between *C. burnetii* seropositivity and number of years lived on a farm (p = 0.028, spline 2 d.f p = 0.586) for students who ever lived on a farm (n = 100). Open circles represent the 95% upper and lower confidence limits.

In the univariate analyses we identified variables associated with *C. burnetii* seropositivity as shown in Table 4. Male students were more often seropositive than females and seropositivity increased significantly with age per year. The study phase, study direction and whether or not internships were followed, were also associated. Moreover contact with cows, pigs, dogs and sheep was positively associated with seropositivity. Students who had lived on a farm were 2.9 times more likely to have *C. burnetii* antibodies. The risk was higher for having lived on a livestock breeding farm and was the highest for a ruminant farm. The risk for a positive serology significantly increased with each year the student had lived on the farm. The shape of this relationship was log-linear, implying that the risk for a positive serology significantly increased with each year the student had lived on the farm (p = 0.028; p-spline 2 df = 0.566; Figure 2). The following activities performed on the farm were associated with seropositivity: animal nursing and work with liquid and/or solid manure. Students reporting to have had a zoonosis during their study had a higher chance of seropositivity. However none of the students reported to have had Q fever during their study (Table 2).General health status and specific clinical symptoms like cough, headache, unusually tired feeling, flu like symptoms and shortness of breath were not associated with seropositivity.

**Table 4:** Univariate analysis of factors possibly associated with seropositivity for *Coxiella burnetii* among veterinary medicine students.

Variable	Odds Ratio (95% CI)	P-value
Male gender (n = 134 (19.9%))	1.74 (1.12–2.73)	0.018^b^
Age (per year)	1.10 (1.05–1.16)	0.000
Study direction farm animals (n = 242 (35.9%))	4.15 (2.76–6.22)	0.000^b^
Zoonotic disease during VM^a^ (n = 132 (19.6%))	2.08 (1.34–3.24)	0.001^b^
Followed VM^a^ internships (n = 171 (25.4%))	2.12 (1.41–3.21)	0.000
*Regular contact with:*		
Horses (n = 451 (66.9%))	1.13 (0.74–1.71)	0.601
Cows (n = 216 (32%))	2.39 (1.60–3.50)	0.000^b^
Pigs (n = 94 (13.9%))	1.72 (1.04–2.85)	0.045^b^
Sheep (n = 192 (28.5%))	1.73 (1.15–2.59)	0.009^b^
Poultry (n = 220 (32.6%))	1.29 (0.86–1.93)	0.246
Goats (n = 166 (24.6%))	1.35 (0.88–2.08)	0.207
Dogs (n = 507 (75.2%))	1.81 (1.10–3.01)	0.022^b^
Cats (n = 496 (73.6%))	0.96 (0.62–1.49)	0.911
Rodents (n = 505 (74.9%))	0.80 (0.52–1.24)	0.362
Birds (n = 283 (42.0%))	1.27 (0.86–1.88)	0.231
Former job with regular animal contact (n = 307 (45.5%))	0.91 (0.62–1.34)	0.692
Ever lived on a farm (n = 100 (14.8%))	2.86 (1.79–4.56)	0.000
Ever lived on a ruminant farm (n = 80 (11.9%))	3.78 (2.30–6.22)	0.000^b^
Ever lived on a livestock breeding farm (n = 67 (10.0%))	3.73 (2.18–6.31)	0.000
Years lived on a farm (per year)	1.07 (1.04–1.10)	0.024
*Activities performed on the livestock farm:*		
Animal nursing (n = 73 (82.0%))	4.40 (1.20–16.14)	0.022
Work with liquid and/or dry manure (n = 61 (68.5%))	3.23 (1.23–8.43)	0.017
Work with straw/hay (n = 75 (84.3%))	3.20 (0.86–11.94)	0.102
Plant nursing (n = 33 (37.1%))	1.61 (0.70–3.71)	0.291
*Compared to currently in study phase 1*		
Currently in study phase 2 (n = 336 (49.9%))	2.20 (1.34–3.62)	0.001^b^
Currently in study phase 3 (n = 117 (17.4%))	2.95 (1.64–5.34)	0.001^b^
*Compared to town (15.000 to 80.000 inh) in childhood*		
Grew up in a village (<15.000 inhabitants) (n = 282 (41.8%))	1.49 (0.97–2.29)	0.183
Grew up in a city (>80.000 inhabitants) (n = 110 (16.3%))	1.28 (0.72–2.27)	0.183
*Compared to currently living in a student house*		
Private house (n = 169 (25.1%))	1.45 (0.94–2.25))	0.218
Parental house n = 71 (10.5%))	0.95 (0.49–1.86)	0.218
*Compared to a none smoker*		
Past smoker (n = 60 (8.9%))	1.11 (0.57–2.17)	0.898
Current smoker (n = 69 (10.2%))	1.13 (0.61–2.12)	0.898

Note.

a VM, veterinary medicine.

b Variables included in the multivariate analysis, other variables p<0.20 were excluded because of multicollinearity.

Ten variables were included in the initial multivariate regression model. In the final model the following were identified to be associated with seropositivity: having lived on a ruminant farm (OR 2.7), study direction ‘farm animals’ (OR 3.3), having had a zoonotic disease during study (OR 1.7) and duration of study (phase 2 (OR 1.8) and phase 3 (OR 2.3), (Table 5)).

**Table 5:** Factors associated with *Coxiella burnetii* seropositivity obtained by multivariate analysis for all students and stratified by ever lived on a farm.

	All	Ever lived on a farm	
	OR (95% CI)	Yes (OR (95% CI))	No (OR (95% CI))
Study direction			
Farm animal health	3.27 (2.14–5.02)	4.86 (1.54–15.29)	3.32 (2.06–5.35)
Other direction	1.00	1.00	1.00
Study phase			
Phase 3 (Year 6)	2.31 (1.22–4.39)	0.43 (0.07–2.66)	3.16 (1.55–6.46)
Phase 2 (Year 3–5)	1.83 (1.07–3.10)	1.34 (0.46–3.94)	2.03 (1.09–3.79)
Phase 1 (Year 1–2)	1.00	1.00	1.00
Zoonotic disease during VM^a^			
Yes	1.74 (1.07–2.82)	7.23 (1.74–30.09)	1.34 (0.78–2.34)
No	1.00	1.00	1.00
Ever lived on ruminant farm			
Yes	2.73 (1.59–4.67)	-	-
No	1.00	-	-
Childhood municipality			
Village	-	0.53 (0.18–1.52)	1.53 (0.89–2.62)
City	-	-	2.18 (1.15–4.14)
Town		1.00	1.00

Note. Multivariate analysis for all students obtained with Forward and Backward logistic regression.

Stratified analysis obtained with Enter.

a VM, veterinary medicine.

We performed stratified analyses for students who had lived on a farm and those who did not, to investigate whether study direction remained an independent risk factor (Table 5). Results showed that the study direction ‘farm animals’ remained significantly associated with seropositivity for those who grew up on a farm (OR study direction = 4.9), as well as for those who did not (OR study direction = 3.3).

## Discussion

In this cross-sectional study among Dutch veterinary students, we found a *C. burnetii* seroprevalence of 18.7% and identified several associated risk factors including study related factors. Only few studies have assessed zoonotic risks for veterinary medicine students. This is the first large-scale study that examined the seroprevalence for *Coxiella* among veterinary medicine students of all study years and directions. The overall observed seroprevalence was within the range of 10 to 40% reported in other studies for veterinary students of Spain, Brazil, California and Ohio [15]–[17].

The found prevalence is considerably lower than the prevalence of over 80% in Dutch livestock veterinarians sampled in 2009 [12]. The prevalence among these veterinarians might be slightly higher than when sampling would have taken place in 2006, due to the environmental outbreaks starting in 2007. Conversely, other studies reported high seroprevalences of 20% and more for veterinarians in countries like the United States, Canada, Slovakia and Taiwan [13], [14], [19]–[21]. Comparing seroprevalences should however be done with caution, because different study populations and diagnostic tests applied might affect the outcomes. Recently, commercial IFAs and ELISAs have become available which are now predominantly used [22]. Despite this progress, there is still a wide interlaboratory variability due to different IgG and IgM cut-off levels applied [22]. There is no general consensus of the appropriate cut-off level as it depends on the population under study and the used antigen-preparation [23]. In this study IFA was used instead of ELISA because it is considered to be the reference method to study seroprevalence of *Coxiella* [24]. We chose a cut-off level of 1∶32 instead of the 1∶16 cut-off recommended by the manufacturer to increase specificity thus lowering the chance of false positives.

We found that students who grew up on a farm, especially on a ruminant farm, had a higher risk of being seropositive. All kinds of animals can be affected by *Coxiella* but ruminants are the most important reservoirs [25]. Furthermore almost all students performed at least one activity on the farm on which they had lived, for example more than 80% performed animal nursing. The shedding of *Coxiella* occurs primarily during aborting or parturition, thus likely occasions whereby students were often present [26], [27]. A study in Spain among veterinary students documented working with ruminants as a risk factor and in Taiwan goat exposure was a risk factor for veterinarians [16], [21].

The risk for a positive serology was found to significantly increase with each year the student had lived on a farm. The biological meaning of this is not known, as profound studies concerning exposure-response relations for *Coxiella* are lacking. Our finding might just reflect the increased probability to encounter *C. burnetii* exposure, as the risk for each exposure moment is constant given that one *Coxiella* organism entering the body is enough to cause disease [1]. On the other hand, our finding might be explained by a cumulative effect of long term exposure, suggesting that a threshold exposure should be met. Lastly, the level of exposure might be of importance as well: the persons who lived longer on a farm are more likely to have performed activities like animal nursing.

Students within the ‘farm animals’ direction had a three times higher risk to be seropositive than students from other directions. The ‘farm animal’ direction itself includes regular contact with ruminants, but ‘farm animal’ students also often had contact with ruminants before or beside their study (Table 3). Furthermore the percentage of students with a farm childhood in this direction is considerably higher. Stratified analyses on farm childhood however showed study direction to be a risk factor also for those with a farm childhood, suggesting two independent effects, indicating also for these students the importance of their study for the development of seropositivity.

Longer study duration was associated with an increased likelihood for seropositivity. As mentioned before, the study has an increasing amount of practical lessons from the second study phase and onwards. Furthermore the last studyphase consists solely of internships whereby largely all veterinary activities are performed by the students. Thus, towards the end of the study the number of animal contact increases as well as the number of treatments executed. The treatment of cattle, swine and wildlife were previously reported as a risk factor for veterinarians [13]. Presumably, treatment of these species by students in their last phase can partly explain studyphase being a risk factor. In addition, by default students in later study phases are older likewise their possibility of becoming infected during their lifetime is higher [9]. Age as a risk factor was also found in a study amongst a Canadian general population and among U.S. veterinarians [13], [19]. It could be argued that students in higher study phases have lived longer on a farm, and therefore are more likely to become seropositive. However, the average number of years students lived on a farm in study phase 1, 2 and 3 did not differ, being respectively 15.03, 14.84 and 16.75 years.

Students reporting zoonoses since the start of their study were more likely to be seropositive, although none of the 960 students reported to have had Q fever. Of the students 20% reported a zoonosis; most prevalent were ringworm and other fungal infections. A variety of fungi are known to be commensals of the animal skin, occasionally they can also be pathogenic either for animals or humans [28]. Students with frequent animal contact are presumably more exposed to several zoonotic pathogens [29]. Good hygiene is important for the prevention of these zoonoses [30]. Presumably zoonotic diseases were found to be a risk factor for *Coxiella* seropositivity because it reflects the students' amount of animal contact and hygiene practices. Whitney *et al* examined the use of personal protective equipment by veterinarians, whereby wearing always a lab coat and always a surgical mask were protective factors [13]. These findings indicate the probable benefit of strict hygienic measures. In contrast, recent findings among culling workers showed seroconversion in around one out of five workers despite the use of personal protective equipment [31].

The seroprevalence of 18.7% for the Dutch veterinary students is high when compared to the seroprevalence of 2.4% for the general population in the Netherlands measured in the same time period, using the same methodology [11]. This indicates *C. burnetii* as a study or occupation related risk for veterinary students, as it also exists for veterinarians. It should be noted that 18.7% is the average prevalence in the study population. The risk for students in certain subgroups is considerably higher. For example the seroprevalence is 37.3% among students in the third study phase within the ‘farm animals’ direction. This overall prevalence of 18.7% is presumably a valid estimate for the general veterinary medicine student population, since about half of the total population provided a blood sample. The students who provided a blood sample showed to be only marginally different from the student population who did not.

The measurement series in the Netherlands revealed that the seroprevalence of students lies in between the prevalence observed in the general population and among veterinarians. However, students at the start of their study already had an increased seroprevalence of 10.9%. These students only have had theoretical courses; hence the increased seroprevalence can only be explained by other determinants, such as the frequent occurrence of a farm childhood in this population and the degree of ruminant contact prior to the start of their study. As could be expected, veterinary students have always been highly interested in animals. A large number of the students had regular contact with different animal species in childhood and around half of the students reported to have had a job with regular animal contact (Table 1). Students in the first phase within the ‘farm animals’ direction had a substantial higher seroprevalence (23.8%) than students in the ‘individually kept animals’ direction (5.7%, Table 3). This is likely a result of previous contact with ruminants, as students with a farm childhood are more likely to choose for the ‘farm animals’ direction.

The risk factors identified comprised most of the risk factors found by several other studies both in open population and occupational settings. However, some other risk factors have been reported before, but could not be studied as the questionnaire did not include these items. An example is contact with pond water and knowledge of Q fever [13], [21].

The implications of the high occurrence rate of seropositivity on students' health are not yet known. None of the students reported to have had Q fever. Q fever has a wide variety of non-specific symptoms and is often asymptomatic, so it is difficult to collect relevant information with a questionnaire over an extended period of time [2], [3]. Poor recall might also have contributed to the low reported prevalence for Q fever. Furthermore the questionnaire was primarily based on the European Community Respiratory Health Survey questionnaire, and was not specifically directed to identify acute Q fever symptoms [32]. On the other hand, a high prevalence of self reported Q fever was not expected as as approximately 60% of Q fever infections are considered to be asymptomatic [4]. Both symptomatic and asymptomatic Q fever has been described to develop into chronic Q fever, although most information is available from symptomatic acute Q fever patients [3].Therefore research is needed to explore the risk for asymptomatic seroconverters of development into chronic Q fever.

This study raises the question whether specific measures have to be taken in this population to prevent development of *C. burnetii* infection. General protective measures may not be sufficient to protect students throughout their career. Therefore offering vaccination may be considered, like in Australia for personnel with high risk occupations [33], or yearly serological screenings as suggested for wool workers [34]. Moreover, in general, awareness about study related health risks should be strengthened. Knowledge regarding clinical symptoms of Q fever can improve referral to the occupational physician affiliated to the university and prevent development of chronic stages of disease.

To conclude, this is the first large-scale study that examined the seroprevalence for *C. burnetii* among veterinary medicine students across all study phases. It demonstrates a considerable *C. burnetii* seroprevalence among veterinary medicine students. Besides regular contact to ruminants outside the curriculum program, also study related factors were associated with seropositivity. This suggests the importance of Q fever as an occupational risk for veterinary medicine students. Interestingly, we demonstrated a log-linear relationship between the numbers of years lived on a farm and seropositivity. Since clinical Q fever illness was not self-reported further research is recommended to study the health implications of seropositivity. Overall, this study contributes to the knowledge and the awareness of Q fever as a risk for veterinary students in order to contribute to its prevention.
